# Interaction between behavioral inhibition and neural alcohol cue-reactivity in ADHD and alcohol use disorder

**DOI:** 10.1007/s00213-020-05492-1

**Published:** 2020-04-13

**Authors:** S Vollstädt-Klein, S Gerhardt, A Lee, A Strosche, G Sharafi, R Nuriyeva, J Seidt, O Hennig, B Alm, D Hermann, WH Sommer, F Kiefer, M Luderer, E Sobanski

**Affiliations:** 1 Department of Addictive Behavior and Addiction Medicine, Central Institute of Mental Health, Medical Faculty of Mannheim, University of Heidelberg, Mannheim, Germany; 2 Children’s Center, Klinikum Frankfurt Oder, Frankfurt (Oder), Germany; 3 Department of Obstetrics and Gynecology, University Hospital, Goethe University, Frankfurt, Germany; 4 Department of Psychiatry and Psychotherapy, Central Institute of Mental Health, Medical Faculty of Mannheim, University of Heidelberg, Mannheim, Germany; 5 Institute of Psychopharmacology, Central Institute of Mental Health, Medical Faculty of Mannheim, University of Heidelberg, Mannheim, Germany; 6 Department of Psychiatry, Psychosomatic Medicine and Psychotherapy, University Hospital, Goethe University, Frankfurt, Germany; 7 Department of Child and Adolescent Psychiatry, University Medical Center Mainz, Mainz, Germany

**Keywords:** Alcohol use disorder, Attention-deficit / hyperactivity disorder, Comorbidity, Functional magnetic resonance imaging, Interference inhibition, Inhibitory control, Impulsivity, Cue-reactivity, Reward system

## Abstract

**Rationale:**

Compared to the general population, adult Attention-Deficit / Hyperactivity Disorder (ADHD) is more prevalent in patients with Alcohol Use Disorder (AUD). Impaired behavioral inhibition is a common characteristic in both ADHD and AUD. Relapse risk is increased in patients with AUD and comorbid, untreated ADHD and in AUD patients with increased neural cue-reactivity.

**Objectives:**

In this study, we examined the interaction between neural correlates of behavioral inhibition and alcohol cue-reactivity with a hybrid imaging task.

**Methods:**

Out of 69 adult study participants, we included *n* = 49 in our final analyses: Individuals had a diagnosis of either AUD (*n* = 13), ADHD (*n* = 14) or both (*n* = 5), or were healthy controls (HC; *n* = 17). The functional magnetic resonance imaging paradigm aimed to examine the combined effects of both an interference-inhibition task (“Simon-task”) and an alcohol cue-reactivity task. Instead of segregating by diagnostic group, we pursued a dimensional approach in which we compared measures of AUD and ADHD severity, as well as the interaction of both, using multiple regression analyses.

**Results:**

The four groups did not differ on the behavioral level on either the inhibition task or the alcohol cue-reactivity task. However, brain activation in frontal control and reward-related regions during completion of the combined tasks were related to ADHD and AUD severity (symptom load). During presentation of both alcohol cues and the inhibition task, participants with higher AUD and ADHD symptom load exhibited greater BOLD (blood oxygen level dependent) responses in subcortical reward-related regions.

**Conclusions:**

Our findings support the hypothesis that ADHD additionally diminishes inhibition ability in individuals with AUD. This may increase relapse risk when confronted with alcohol cues. Further, it is crucial for patients with comorbid AUD and ADHD to take into account not only reduced cognitive control over behavioral inhibition but also simultaneously heightened alcohol cue-reactivity.

**Electronic supplementary material:**

The online version of this article (10.1007/s00213-020-05492-1) contains supplementary material, which is available to authorized users.

## Introduction

Attention-Deficit/ Hyperactivity Disorder (ADHD) is a common childhood onset mental health disorder persisting in many cases until adulthood (Huntley et al., 2012). The worldwide prevalence of adult ADHD is estimated to be 2.8% in the general population (Fayyad et al., 2017). ADHD increases the risk for heavy substance use or developing a substance use disorder (SUD), particularly alcohol use disorder (AUD; Charach, Yeung, Climans, & Lillie, 2011; Estévez-Lamorte et al., 2019; Estévez et al., 2015; Lee, Humphreys, Flory, Liu, & Glass, 2011; Vogel et al., 2016; Wilens & Morrison, 2011). Within the AUD population, prevalence of adult ADHD ranges from 7.7% to 21.1%, with a rate of 20.5% in a German sample (Daigre et al., 2015; Luderer et al., 2018; Reyes et al., 2016; Roncero et al., 2019). The increased prevalence of ADHD in adults with AUD seems to be greatly attributable to ADHD individuals being more susceptible to early alcohol use, a persisting risky drinking behavior and is predictive of the maintenance of AUD in later life (Charach et al., 2011; Estévez-Lamorte et al., 2019; Estévez et al., 2015; Lee et al., 2011; Wilens & Morrison, 2011). The co-occurrence of these disorders might be due to shared genetics (Capusan, Bendtsen, Marteinsdottir, Kuja-Halkola, & Larsson, 2015; Edwards & Kendler, 2012) or neuropsychological factors such as increased impulsivity (Pedersen et al., 2016; Roberts, Peters, Adams, Lynam, & Milich, 2014) or decreased inhibitory control (Smith, Mattick, Jamadar, & Iredale, 2014).

Surveying past neuroimaging studies examining impulsivity, inhibitory control and reward processing in healthy individuals, certain brain regions seem to stand out: the anterior cingulate cortex (ACC), the prefrontal cortex (PFC), motor regions, the angular gyrus (AG) and subcortical regions like the insula and the striatum all seem to be involved (Wager et al., 2005). The ACC has mostly been associated with conflict monitoring (Botvinick, Braver, Barch, Carter, & Cohen, 2001; Egner & Hirsch, 2005) and is theorized to send signals to the cognitive control system with its seed in the PFC, more specifically the inferior frontal gyrus (IFG; Egner & Hirsch, 2005; Munakata et al., 2011; Tabibnia et al., 2011). Studies in non-clinical individuals on response inhibition using different paradigms including the go/no-go task have supported the involvement of the PFC and ACC (Chikazoe, Konishi, Asari, Jimura, & Miyashita, 2007; Fan, Flombaum, McCandliss, Thomas, & Posner, 2003; Menon, Adleman, White, Glover, & Reiss, 2001). As part of the mesolimbic reward system, the striatum has not only been analyzed extensively regarding inhibitory control but also regarding SUD. In the context of impulsivity, the striatum has been often associated with reward deficiency, which is further underscored by neurobiological findings: it has been suggested that a lack of D2 dopamine receptors in the neural reward system due to genetic variations predisposes for multiple addictive, impulsive and compulsive behaviors (Blum et al., 2000; Bowirrat & Oscar-Berman, 2005). The altered dopamine signaling consequently affects reward-related activation of the ventral striatum (VS), which has been widely associated with reward processing (Forbes et al., 2009). A novel study in which post-mortem brains of individuals with AUD were examined and integrated with results from experiments on alcohol-dependent rats found a convergent dopaminergic story – in both cases the mesolimbic dopamine system, including D1 receptors and dopamine transporter, appeared to be dynamically regulated over time and evidenced differing characteristic states during withdrawal, abstinence, protracted abstinence and relapse. In this study, Hirth et al. (2016) found a hyperdopaminergic state associated with protracted abstinence, which has in other contexts also been associated with a risk for increased impulsivity and eventual relapse. A recent review has generated further support for this theory of dynamic regulation and change in striatal dopaminergic signaling over the course of the addiction life-cycle, highlighting a potential source for the variability in findings on dopaminergic signaling in addiction to date (Hansson et al., 2019). A negative association, i.e. a correlation of low VS activation during reward anticipation with high levels of impulsivity, has been observed in AUD patients but not in healthy controls (Beck et al., 2009). Interestingly, other studies suggest that this relationship between inhibition and reward sensitivity might not simply be a consequence of AUD, but a causal driver. Examining neural correlates of inhibition and reward in healthy individuals, Weafer, Crane, Gorka, Phan, and de Wit (2019) observed a negative correlation between brain activation during an inhibition task in right prefrontal areas (inferior frontal gyrus, middle frontal gyrus, supplementary motor area) and activation in the left VS during a monetary reward task (Weafer et al., 2019). Decreased prefrontal inhibition was associated with increased VS-mediated reward sensitivity even prior to the development of any SUD, suggesting that pre-existing differences in the fronto-striatal pathways of healthy individuals could predispose to the development of certain disorders.

As already mentioned, impulsivity and deficits in inhibitory control are hallmarks of both ADHD and SUD (Barkley, 1997; de Wit, 2009; Herman & Duka, 2019; Pedersen et al., 2016; Rubio et al., 2008; Smith et al., 2014; Wright, Lipszyc, Dupuis, Thayapararajah, & Schachar, 2014). It has been suggested that impulsivity and deficits in inhibitory control mediate in part the relationship between a preceding ADHD diagnosis and the later development or maintenance of AUD, while also inherent to both disorders separately (de Wit, 2009; Egan, Dawson, & Wymbs, 2017; Pedersen et al., 2016; Roberts et al., 2014; Rubio et al., 2008).

Meta-analyses of neuroimaging studies in patients with ADHD report hypoactivation of several brain regions, including the PFC. During interference inhibition tasks individuals with adult ADHD showed reduced activation in the right inferior frontal cortex, supplementary motor area, ACC, left posterior parietal lobule, insula and dorsal striatum (DS; Hart, Radua, Nakao, Mataix-Cols, & Rubia, 2013). Imaging studies on the reward system in ADHD observed hypoactivation of the VS (Plichta & Scheres, 2014; Plichta et al., 2009), part of the well-known reward circuitry that also encompasses the DS, thalamus, hippocampus, amygdala, insula and dorsolateral and inferior frontal cortices (with orbital, medial and cingulate regions) (Bechara, 2005; Goldstein & Volkow, 2002; Koob & Volkow, 2010). This stands in opposition to findings in AUD patients, where hyperactivation of the VS in response to alcohol cues is observed. With respect to the dorsal striatum and amygdala, however, Plichta and Scheres (2014) report hyperactivation in a reward related delay discounting task (Plichta et al., 2009).

In the development and maintenance of AUD, several brain networks play a role – with an emphasis on limbic circuits and frontal areas (Goldstein & Volkow, 2002). The cue-reactivity construct has gained increasing clinical relevance as it has been demonstrated to evoke drug-like responses on a subjective (e.g. subjective craving), behavioral (e.g. drug-seeking), and physiological (e.g. change of heart rate) level and responses were shown to be associated with compulsive drug use (Grüsser et al., 2004; Weiss et al., 2001) and self-reported craving intensity (Smolka et al., 2006). Neuroimaging studies have revealed, amongst other findings, a shift from the ventral to the dorsal striatum in neural activation. This has been observed when comparing light social drinkers to heavy drinkers after instructing them to look at alcohol-related pictures (Vollstädt-Klein et al., 2010). Neural cue-reactivity has been shown to also predict treatment outcome: examining functional magnetic resonance imaging (fMRI) activation patterns as prognostic factors for relapse in in-house patients with AUD, Reinhard et al. (2015) observed increased cue-induced activation in the VS and the orbitofrontal cortex (OFC). The association between increased cue reactivity within the striatum and later relapse risk was further examined using pharmacotherapy in individuals with AUD. Naltrexone, a opioid antagonist, was most effective in individuals with high cue reactivity within the DS and further reduced the relapse risk during the first three months of abstinence (Bach et al., 2020). It has also been observed that individuals, who drink primarily for the rewarding effects of alcohol, benefitted the most from a treatment with Naltrexone (Witkiewitz, Roos, Mann, & Kranzler, 2019). A meta-analysis examining functional neuroimaging findings of alcohol cue-reactivity (Schacht, Anton, & Myrick, 2013) revealed further robust neural activation patterns in limbic (VS) and prefrontal regions (ACC, ventromedial PFC), parietal (posterior cingulate cortex (PCC) and precuneus) and temporal regions (superior temporal gyrus). In general, individuals with drug addictions have been observed to show hyperactivation not only in the reward, but also in salience, habit, memory and executive control networks during drug cue exposure across several types of addiction (Zilverstand, Huang, Alia-Klein, & Goldstein, 2018). Therefore, a greater neural response during drug cue-related tasks can be observed in several brain regions: VS, ACC, OFC and anterior PFC (reward network); putamen and caudate (habit network); insula, dorsal ACC and inferior parietal lobule (salience network); ventrolateral PFC and dorsolateral PFC (executive network); dorsomedial PFC, PCC and precuneus (self-direction network); hippocampus and parahippocampus (memory network) (Zilverstand et al., 2018). Interestingly, when it comes to inhibitory and non-drug related tasks, hypoactivation of some of these same control, executive and reward related regions have been observed in patients with AUD (Luijten et al., 2014; Spechler et al., 2016; Zilverstand et al., 2018).

In sum, overlapping neural circuitries play a role in the imbalance between impaired inhibition and increased reward functioning (Goldstein & Volkow, 2002). Combining neuroimaging findings on impulsivity and cue-reactivity with the high prevalence of comorbid AUD and ADHD diagnoses raises the question whether people with both disorders show significantly more pronounced impairments in neural correlates of inhibitory control and/ or elicit a stronger neural alcohol cue-reactivity than those with neither or only one of these disorders. Though a direct comparison between diagnostic groups is possible, we instead opted to pursue a dimensional approach in addressing this question for several reasons. A primary concern was the inherent limited sample size of comorbid AUD + ADHD patients. These patients are not only hard to recruit based on exclusion criteria, such as current psychoactive medication, but are also simply harder to find and data obtained from these individuals is often not useable due to quality issues, such as exceeding allowed movement parameters in the MRI scanner, for example. Most importantly, however, we decided to analyze our data according to symptom load instead of diagnosis based on the fact that this dimensional approach more closely reflects the current scientific understanding of mental disorders (e.g. National Institute of Mental Health – Research Domain Criteria (NIH RDoC)). A binary assignment to ‘healthy’ or ‘diseased’ categories has been supplanted by the notion that dysfunction exists along a continuum of severity and across multiple functional domains. We believe that viewing data through this lens allows for a more nuanced and granular analysis of the underlying relationships between neural activity and clinical manifestation.

In this study, we examined the interaction between response inhibition and cue-reactivity using a hybrid imaging task in which individuals were confronted with alcohol stimuli as distractors while performing a response inhibition task. By combining a traditional cue-reactivity task with an interference inhibition task, main effects of both cue-reactivity and inhibitory control can be estimated, as well as any potential interaction effects. In looking at 1) AUD-only individuals, 2) ADHD-only individuals, 3) AUD + ADHD individuals and 4) healthy controls, we expected measures of response inhibition to correspond significantly with ADHD severity and cue reactivity to correspond significantly with AUD severity. Furthermore, we hypothesized that cue-type (alcohol vs. neutral) would have an effect on measures of response inhibition and differentially affect the 4 groups: we expected individuals with both AUD and ADHD diagnoses to show the most pronounced inhibitory deficits upon alcohol cue presentation. In other words, we hypothesized the existence of an additive interaction effect between the axes of ADHD and AUD severity. Individuals with high symptom load in both domains should show more significant impairments than individuals with high symptom load in only one domain on both the behavioral and neural level. We expected a positive relationship between subcortical cue-reactivity and AUD severity that becomes more pronounced the higher the concurrent ADHD symptom load. By contrast, we expected to observe a negative relationship between prefrontal brain activity and ADHD severity that becomes more pronounced the higher the concurrent AUD symptom load.

## Materials and methods

### Participants

A total of 69 subjects participated in the study from October 2014 to June 2017. Individuals had a diagnosis of either AUD (*n* = 13), ADHD (*n* = 14) or both (*n* = 5), or were healthy controls (HC; *n* = 17). For the AUD group, individuals with a diagnosis of an at least moderate AUD (following the Diagnostic and Statistical Manual of Mental Disorders fifth version (DSM-5; American Psychiatric Organisation, 2013)) were included, which corresponds to former DSM-IV nomenclature “dependence” (Dawson, Goldstein, & Grant, 2012). Detailed sample characteristics of the final sample are displayed in Table 1. Requirements for participation were age between 18 and 66 years and normal or corrected-to-normal vision (binocular visual acuity ≥0.8). Exclusion criteria were current use of psychotropic or anticonvulsive medications, epilepsy, cirrhosis of the liver, suicidal tendencies, severe neurological or medical illness or any MRI-exclusion criteria (e.g. metal implants, pacemakers, epilepsy, pregnancy). Individuals belonging to the groups AUD or AUD + ADHD showed no other substance use disorders apart from alcohol or nicotine according to the DSM-5 (World Health Organization, 2004). Further, they had to be abstinent for at least five days prior to study inclusion and remained abstinent for the time of participation. A medically supervised detoxification (treatment of withdrawal symptoms with short-acting benzodiazepines) had to have been completed for at least 3 days. Individuals belonging to the groups ADHD or AUD + ADHD had a diagnosed adult ADHD according to the DSM-5 without receiving any ADHD-specific medication. For more detailed inclusion and exclusion criteria see supplementary Table 1. All patients were recruited from the clinic of the Department of Addictive Behavior and Addiction Medicine and the Psychiatric Outpatient Clinic of the Clinic for Psychiatry and Psychotherapy, both located at the Central Institute of Mental Health (Mannheim). Healthy volunteers were recruited through public announcements (newspaper advertisements, flyers, social media) or lists of subjects who had already participated in a study at the institute and who gave written consent about being contacted for further studies. The Ethics Committee of the Medical Faculty Mannheim, Heidelberg University, approved the study (approval number 2013-530 N-MA). In accordance with the Declaration of Helsinki, all participants provided written informed consent.

**Table 1:** Mean (SD) group characteristics of all participants (*N* = 49)

	**AUD**	**ADHD**	**AUD + ADHD**	**Control**	**ANOVA**^**a**^**/ Welch**^**b**^
**N**	13	14	5	17	
**Male:Female**	13:0	13:0	5:0	11:6	χ2(3) = 7.39, *p* = 0.061
**Age [years; mean(SD)]**	45.4 (12.3)	32.2 (10.6)	41.2 (9.5)	39.2 (12.7)	F(3,43) = 2.709, *p* = 0.057^a^
**Smoker** **[yes:no:unknown]**	8:3:2	2:10:2	4:1 2	13:2	χ2(3) = 15.67, p < 0.001
**AUQ [mean(SD)]**	12.2 (6.8)	8.8 (1.7)	14.0 (5.1)	9.9 (3.3)	F(3,14) = 2.576, *p* = 0.095^b^
**AUDIT [mean(SD)]**	27.6 (5.0)^1,2^	2.5 (2.7)^1,3^	18.6 (8.3)^3,4^	3.2 (2.4)^2,4^	F(3,14) = 89.450, p < 0.001^b^
**ADS [mean(SD)]**	16.2 (5.6)^1,2^	2.2 (3.2)^1,3^	11.8 (5.3)^3,4^	2.5 (2.9)^2,4^	F(3,14) = 23.222, p < 0.001^b^
**WURS-k [mean(SD)]**					
**Attention deficits/hyperactivity**	5.8 (5.5)^1,2^	16.4 (5.6)^1,3^	23.8 (5.8)^2,4^	5.0 (5.3)^3,4^	F(3,43) = 23.271, p < 0.001^a^
**Impulsivity**	1.7 (1.9)^1,2^	5.6 (4.3)^1,3,4^	12.2 (1.3)^2,3,5^	1.3 (1.8)^4,5^	F(3,18) = 77.565, p < 0.001^b^
**ADHD-SR [mean(SD)]**					
**Attention deficits**	2.7 (2.9)^1,2^	18.2 (4.0)^1,3^	19.4 (4.2)^2,4^	2.7 (2.5)^3,4^	F(3,44) = 89.668, p < 0.001^a^
**Hyperactivity**	1.8 (2.8)^1^	5.6 (5.1)^2,3^	11.4 (2.3)^1,3,4^	0.5 (0.7)^2,4^	F(3,13) = 37.286, p < 0.001^b^
**Impulsivity**	1.3 (1.6)^1,2^	5.4 (3.3)^1,3^	8.0 (3.5)^2,4^	0.8 (1.0)^3,4^	F(3,13) = 13.236, p < 0.001^b^
**Overall score**	5.8 (5.2)^1,2^	29.3 (7.2)^2,3^	38.8 (8.9)^2,4^	4.0 (3.5)^3,4^	F(3,14) = 62.812, p < 0.001^b^

Note: AUQ = Alcohol Urge Questionnaire; AUDIT = Alcohol Use Disorder Identification Test; ADS = Alcohol Dependence Scale; WURS-k = German short version of the Wender-Utah-Rating-Scale; ADHD-SR = ADHD self-report scale. Superscript letters and symbols indicate the statistical test used for group comparisons (ANOVA^a^/ Welch^b^) and describe significant post-hoc test results (^1 2 3 4^; *p* < 0.05) with respect to the group

### Procedure

First, all individuals had to undergo a screening procedure for AUD and ADHD (Alcohol Use Disorder Identification Test (AUDIT; Reinert & Allen, 2002), cut-off <8 for HC and ADHD; Wender-Utah-Rating-Scale (WURS-k; Rösler et al., 2008) for HC and AUD, cut-off <30 for HC and AUD; ADHD self-report scale (ADHD-SR; Rösler et al., 2008) for HC and AUD, cut-off <6 for items one to nine and cut-off <6 for items 10 to 18; Adult ADHD Self-Report Scale (ASRS-SR; Kessler et al., 2005) for HC and AUD, cut-off <14). According to the results, this screening was then followed by a clinical diagnostic session regarding AUD or ADHD. ADHD was diagnosed clinically according to DSM-5 for the groups ADHD and ADHD+AUD in the Psychiatric Outpatient Clinic of the Clinic for Psychiatry and Psychotherapy by an experienced clinician. Assessment of ADHD included self-report scales and at least one full diagnostic interview, as well as school records and informants’ ratings, if possible. In addition, participants considered ADHD or HC had to be below the clinical cut-off for the AUD screening questionnaire. AUD was diagnosed accordingly in the Department of Addictive Behavior and Addiction Medicine for the groups ADHD and ADHD+AUD by an experienced clinician. In addition, participants considered AUD or HC had to be below the clinical cut-off for a variety of ADHD screening questionnaires. After being included in the study and assigned to one of the four groups, all participants received a battery of questionnaires, which they had to fill out prior to the fMRI experiment. This battery included the Alcohol Dependence Scale (ADS; Skinner & Horn, 1984), the AUDIT (Reinert & Allen, 2002), the Alcohol Urge Questionnaire (AUQ; Bohn, Krahn, & Staehler, 1995), the WURS-k (Retz-Junginger et al., 2002), the ADHD-SR (Rösler et al., 2008), and the Fagerström Test for Nicotine Dependence (FTND; Heatherton, Kozlowski, Frecker, & Fagerstrom, 1991). Furthermore, demographic variables and history of drug consumption were assessed.

### FMRI task

**Fig. 1:**
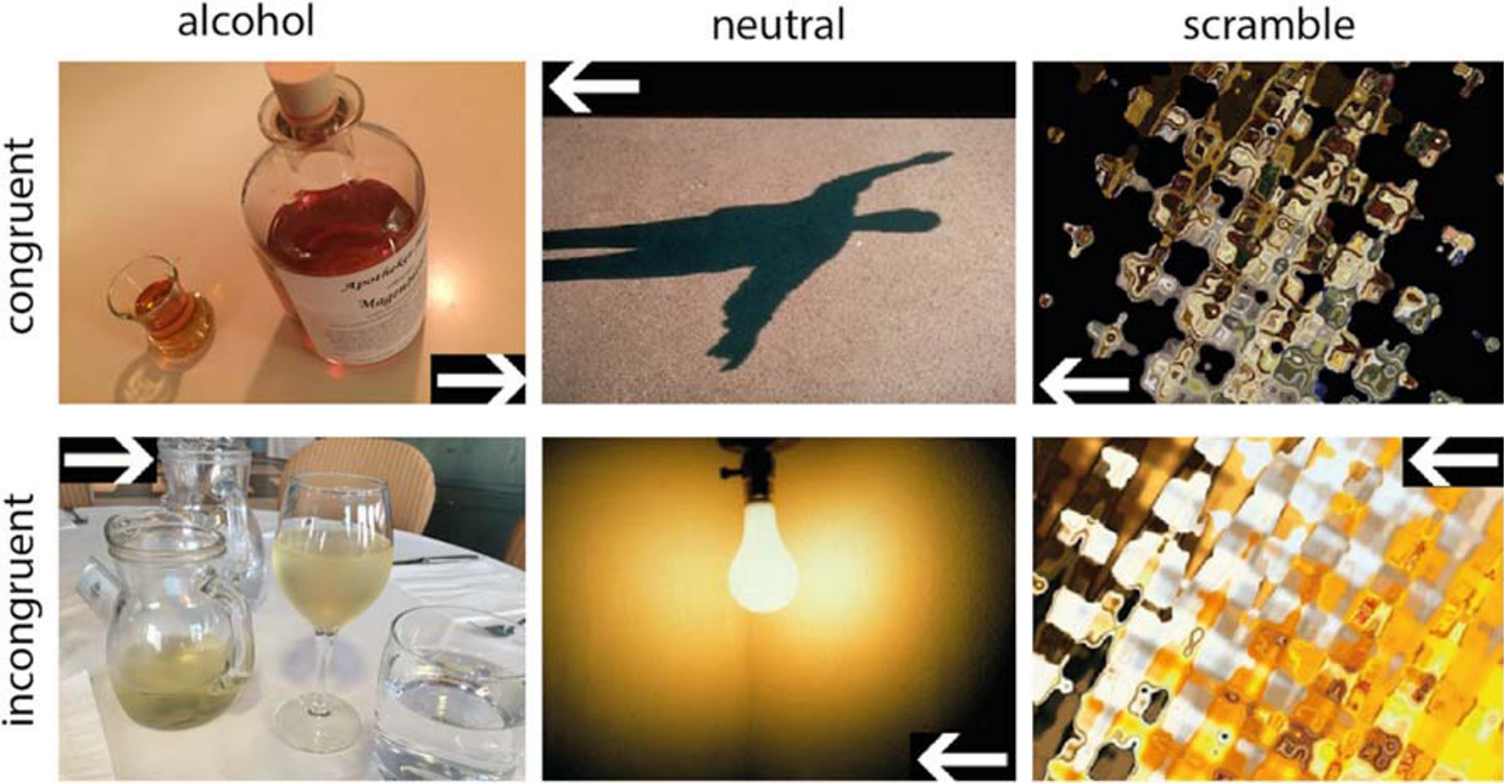
Functional magnetic resonance imaging (fMRI) event-related design including alcohol, neutral International Affective Picture Series (IAPS), and scrambled pictures in the conditions congruent and incongruent; every image was presented for 1500 ms.

The participants’ task was to determine whether a target (a white arrow presented against a black background) pointed to the right or to the left side of the screen. The target appeared in one of four possible locations on the screen (top or bottom, left or right corner). A congruent trial is one in which the arrow appears on the same side of the screen in which it is pointing (e.g. arrow appearing in the top/bottom left corner and pointing to the left side). An incongruent trial is one in which the arrow appears on the opposite side of the screen in which it is pointing (e.g. arrow appearing in the top/bottom left corner and pointing to the right side). The target was displayed on a background that showed either an alcohol image, a neutral image, or a scrambled image. For each picture category (alcohol – neutral – scramble) 30 images were chosen and presented four times throughout the task, once in each of the task conditions (congruent – incongruent, left – right). Every image was presented for 1500 ms. A total of 360 trials was conducted in an event-related design with 90 trials for each of the 2 × 2 conditions [congruency (congruent / incongruent) x side (left /right)]. Participants were asked to press the left button of the arrow keyboard when the target was pointing to the left side of the screen and to press the right button when the target was pointing to the right side. Examples for the task images are displayed in Fig. 1. The order of the image categories (alcohol – neutral – scramble) was pseudorandomized. The presentation of an image within a category, the presentation of the arrow on the top – bottom, and the task condition (congruent – incongruent, left – right) were randomized for each participant. The total duration of the task was 9 min 26 s. Before the experiment in the scanner, participants received a training run on a laptop computer outside the scanner. Participants were also instructed orally to respond as quickly and accurately as possible. As dependent measure the average response time for incongruent - congruent and alcohol – neutral/scramble trials was used.

Images for the alcohol category were chosen from our own alcohol picture series (Vollstädt-Klein et al., 2010), which has been demonstrated to evoke craving in detoxified alcohol-dependent patients (unpublished data). For the neutral category, images were taken from the International Affective Picture Series (IAPS; Lang, Bradley, & Cuthbert, 1999) and were matched for color distribution and complexity to the alcohol cues. To create images for the scramble category, the images chosen for the alcohol category were edited in the program Corel Photo Paint (Corel Corporation, München, Deutschland) using the effect options ‘Ripple’ and ‘Twirl’ to distort the images.

### FMRI acquisition

Scanning was performed using a 3 T whole-body tomograph (MAGNETOM Trio, TIM-technology; Siemens, Erlangen, Germany), which we used to acquire 233 T2*-weighted transversal echo-planar images (TR = 2.41 s, TE = 25 ms, flip angle = 80°, 42 slices, slice thickness: 2 mm, 1 mm gap, voxel dimensions 3 × 3 × 3 mm^3^, FOV 192 × 192 mm^2^, 64 × 64 in-plane resolution) covering the entire brain. A field map acquisition was conducted to correct the fMRI data for geometric distortion caused by magnetic field inhomogeneities (TR = 460 ms, TE = 5.19 ms / 7.65 ms, flip angle = 60°, voxel size = 3 × 3 × 3 mm). Additionally, a 5:21 min anatomical scan was performed to acquire a T1-weighted 3D MPRAGE (Magnetization Prepared- Rapid Gradient Echo) dataset (192 sagittal slices, TR = 2.30s, TE = 3.03 ms, TI = 900 ms, flip angle = 9°, slice thickness: 1 mm, 0.5 mm gap, voxel dimensions 1 × 1 × 1.5 mm^3^, FOV 256 × 256 mm^2^, 256 × 256 in-plane resolution). Images were presented to patients using MRI Audio/Video Systems goggles (Resonance Technology Inc., Los Angeles, CA, USA). Tasks were presented using Presentation® software (Version 16.5, Neurobehavioral Systems, Inc., Albany, CA, USA).

### FMRI preprocessing

Pre-processing and statistical analyses of brain imaging data were performed using SPM8 (Wellcome Trust Centre for Neuroimaging, London, UK). The first five scans were excluded to avoid artifacts caused by magnetic saturation effects. The remaining 228 scans were corrected for residual geometric distortion based on the acquired magnetic field map, spatially realigned to correct for head motion, temporally realigned to minimize temporal differences in slice acquisition and normalized to a template provided by MNI (Montreal Neurological Institute, Quebec, Canada). Subsequent smoothing was performed using an isotropic Gaussian kernel (8 mm FWHM).

### Statistical analysis

Statistical analysis of the pre-processed fMRI data on the first-level was performed by modeling the six different conditions of interest (alcohol, neutral and scramble pictures in congruent / incongruent trials with correct responses) as explanatory variables within the context of the general linear model on a voxel by voxel basis and convoluted with the canonical hemodynamic response function. Also, motion regressors from the preprocessing were included. Incorrect and missing responses were included as regressors of no interest. Furthermore, a quality check was performed. Subjects with excessive head movement (> 3 mm/ 3°) or other artifacts were excluded from the subsequent analysis.

Resulting contrast images of interest from the first-level analysis are the main effects “cue type (“alcohol vs. neutral/scramble”) and congruency (“incongruent vs. congruent”) and their interaction effects (i.e. “alcohol > neutral/scramble, incongruent > congruent” and “alcohol > neutral/scramble, incongruent < congruent”). We used a dimensional approach for each contrast of interest separately instead of segregating groups by diagnosis. For this purpose, the individual contrast images were included in second-level multiple regression analysis to identify brain regions with effects of ADHD symptom load, AUD symptom load and their corresponding interaction. Additionally, we conducted group comparison analysis segregating groups by diagnosis using a full factorial model (see supplementary material). These supplementary analyses were conducted to give further explanations, but should be interpreted with caution due to the small sample size of the combined group (AUD + ADHD).

The ADS sum score was used for assessing AUD severity. The overall ADHD-SR score was used to describe ADHD severity in general. Additionally, the “impulsivity” score of the ADHD-SR was included in an additional analysis due to its relevance with respect to our sample and task. Furthermore, the interaction effect of ADHD and AUD severity was modeled by the term ((ADS - _mean_ADS)*ADHD-SR - _mean_ADHD-SR)) for the overall and impulsivity factor scores of the ADHD-SR separately. Age was included as covariate of no interest in all analyses. To control for multiple statistical testing, the probability for a family-wise error (FWE) was set to 0.05. Using 10,000 Monte Carlo Simulations in AFNI’s 3dClustSim (Analysis of Functional NeuroImages, www.afni.nimh.nih.gov/) a voxel-wise-threshold of *P* < 0.001 in combination with a cluster-extend-threshold of *k* ≥ 151 was determined for the imaging analysis with an automatically conducted estimation of smoothness. For further analyses, we created functional masks in the following manner. We identified regions surviving in all second-level analyses and created spheres of 10 mm diameter around the peak activation of each cluster using WFU_PickAtlas in SPM8. After checking the overlay of mask and clusters, for stretched clusters we created a second sphere around the peak voxel of the largest sub cluster. This approach prevented us from losing relevant regions. From these functional and sphere masks, we created the final functional regions of interest (ROI) as intersection masks around the peak voxels using the image calculator (ImCalc) in SPM8. The resulting masks were used to aggregate contrast values (i.e. weighted beta values) for each subject. We will refer to them as “functional masks”. The anatomical localization of the results was determined using xjView (http://www.alivelearn.net/xjview) and visualized using MRIcro (Rorden & Brett, 2000).

Reaction times (RT), interference effects as well as sample characteristics, questionnaires and aggregated contrast values from the fMRI analyses were analyzed using the Statistical Package for the Social Sciences (SPSS) version 24.0 (IBM Corporation, Armonk, NY, USA). The general interference effect was calculated by subtracting the mean RT of the correct congruent trials from the mean RT of the correct incongruent trials. By comparing this interference effect between neutral/scramble and alcohol trials, the interaction between interference effects and stimulus type can be examined, which corresponds to the distraction by alcohol cues influencing the interference effect. Further, relevant questionnaire scores were calculated and a descriptive analysis of the sample was performed. Analyses of variance (ANOVA) were used to demonstrate possible group differences with respect to both questionnaire scores and behavioral measures. Regarding the main effects of AUD and ADHD symptom load, and the interaction effect of both on RT and interference effects, multiple regression analyses were performed. In order to assess and visualize the behavioral measures (RT and interference effects) and aggregated contrast values from the fMRI analyses against AUD and ADHD symptom load, we performed a median split of the cohort into high and low AUD and high and low ADHD load, respectively. This allows analyzing neural activity and behavioral outcome in high/ low AUD with regard to ADHD symptom load, and vice versa. Further, possible interaction effects of AUD and ADHD severity can be assessed.

## Results

From the overall sample of 69 participants, 49 subjects were included in the fMRI analysis: 13 AUD, 14 ADHD, 17 healthy controls and five AUD + ADHD. Reasons for excluding participants from the final fMRI analysis were incomplete data, heavy movement in the scanner and not meeting inclusion criteria (e.g. positive reporting of drugs and medication, see CONSORT diagram, Fig. 1 in supplementary material). Questionnaire scores and characteristics of the analyzed sample are displayed in Table 1.

### Behavioral data

The ANOVA revealed no significant group differences in RT or interference effects (*p* > .05). Further details are displayed in Table 2A in the supplementary material. The multiple regression analyses revealed no significant results for the main effects of AUD and ADHD severity and the interaction of both on RT and interference effects (*p* > 0.1). Regarding the difference between low and high levels of AUD severity, higher levels of ADHD severity (“impulsivity”) lead to a smaller general interference effect in individuals with low levels of AUD (see Table 2B in supplementary material). Regarding the difference between low and high levels of ADHD severity, higher levels of AUD severity lead to a greater difference in RT for alcohol vs. neutral stimuli in individuals with low levels of ADHD (“overall”) (see Table 2C in supplementary material). No significant results were observed for further behavioral measures, e.g. the interaction between interference effect and stimulus type, or the interaction between high severity in both ADHD and AUD.

**Table 2:** Regions of interest (ROIs) for contrast value aggregation. Seven ROIs resulted from the interaction of AUD and ADHD symptom load. Additionally, five ROIs were created from corresponding subclusters. A combined voxel-wise [*P* < 0.001] and cluster-extent threshold [k > = 151 voxel], corresponding to pFWE <0.05) was used to identify the ROIs

**Mask n°**	**Side**	**Brain Areas**	**F-value of peak coordinate**	**MNI coordinates**
**1**	R	Right middle frontal gyrus	18.97	40 56 18
**2**	R	Sub-lobar regions (Thalamus)	30.82	28–12 18
**2a**	R	Pallidum	20.39	22–10 -4
**3**	R	Middle temporal gyrus	26.49	52–74 18
**4**	R	BA10	25.56	40 58 24
**5**	R	Insula	22.22	32 26 0
**5a** **5b**	R R	Inferior frontal gyrus Putamen	17.03 17.01	46 26 10 24 8 2
**6**	L&R	BA18, lingual gyrus	19.24	-6 -74 -2
**6a**	R	Lingual gyrus	15.61	26–70 3
**7**	R	Superior frontal gyrus	18.63	40 58 20

### FMRI data

**Fig. 2:**
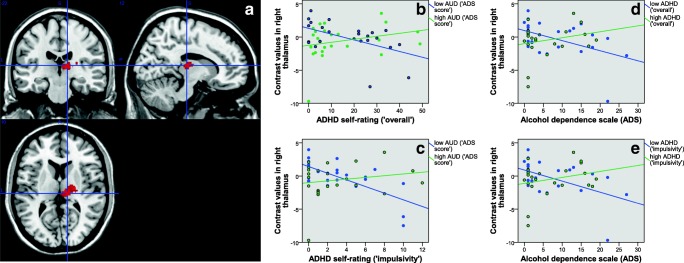
A: functional ROI mask of the right thalamus for contrast value aggregation; B-E: association between AUD or ADHD severity and aggregated brain activation (contrast “alcohol vs. neutral/scramble, incongruent vs. congruent”; *n* = 49); B + C: median split of all participants into low and high AUD, association of brain activation with ADHD severity (B:‘overall’; C:‘impulsivity’); D + E: median split of all participants into low and high ADHD (D:‘overall’; E:‘impulsivity’), association of brain activation with AUD severity

Task main effects (i.e. contrasts “alcohol vs. neutral/scramble” and “incongruent vs. congruent”) were neither dependent on AUD or ADHD severity, nor did they show an interaction effect between AUD and ADHD symptom load. Regarding the interaction of congruency and cue type (i.e. contrast “alcohol vs. neutral/scramble, incongruent vs. congruent”), the analyses resulted in significant clusters for associations between brain activation and AUD severity and also for the interaction of AUD and ADHD severity. No significant results were found for the main effect of ADHD severity. ADS sum score correlated negatively with activation in the left and right precuneus, the inferior parietal lobule (right angular gyrus and left supramarginal gyrus), the left and right cuneus and the left pre- and postcentral gyri (see Fig. 2). Significant interaction effects of ADHD and AUD severity were observed in the right middle temporal gyrus and the right middle frontal gyrus (BA10; see supplementary Fig. 3) but also in right and left lingual gyrus, right insula, putamen, pallidum and thalamus and right superior and inferior frontal gyrus (see supplementary Fig. 4).

**Fig. 3:**
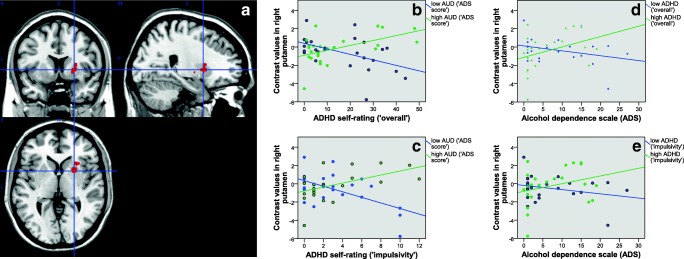
A: functional ROI mask of the right putamen for contrast value aggregation; B-E: association between AUD or ADHD severity and aggregated brain activation (contrast “alcohol vs. neutral/scramble, incongruent vs. congruent”; n = 49); B + C: median split of all participants into low and high AUD, association of brain activation with ADHD severity (B:‘overall’; C:‘impulsivity’); D + E: median split of all participants into low and high ADHD (D:‘overall’; E:‘impulsivity’), association of brain activation with AUD severity

**Fig. 4:**
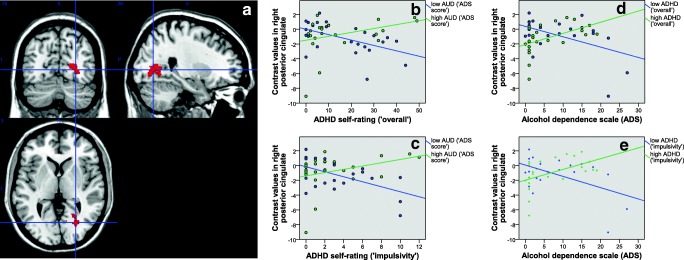
A: functional ROI mask of the right lingual gyrus for contrast value aggregation; B-E: association between AUD or ADHD severity and aggregated brain activation (contrast “alcohol vs. neutral/scramble, incongruent vs. congruent”; n = 49); B + C: median split of all participants into low and high AUD, association of brain activation with ADHD severity (B:‘overall’; C:‘impulsivity’); D + E: median split of all participants into low and high ADHD (D:‘overall’; E:‘impulsivity’), association of brain activation with AUD severity

**Fig. 5:**
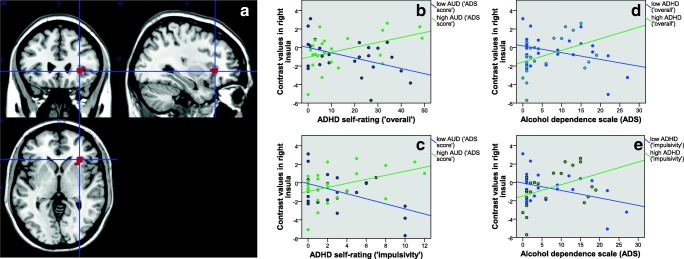
A: functional ROI mask of the right insula for contrast value aggregation; B-E: association between AUD or ADHD severity and aggregated brain activation (contrast “alcohol vs. neutral/scramble, incongruent vs. congruent”; n = 49); B + C: median split of all participants into low and high AUD, association of brain activation with ADHD severity (B:‘overall’; C:‘impulsivity’); D + E: median split of all participants into low and high ADHD (D:‘overall’; E:‘impulsivity’), association of brain activation with AUD severity

For detailed results following extraction of contrast values from several ROIs defined by the aforementioned peak coordinates see Table 2. The results of Pearson correlation analyses between brain activation and ADHD or AUD symptom load in either high or low ADHD/AUD individuals (median group split) are listed in Table 3 and Figs. 2 to 5. Regarding the difference between low and high levels of AUD severity (see Table 3A) interaction effects in the right middle, superior and inferior frontal gyrus, BA10, posterior cingulate and thalamus, and bilateral lingual gyrus were driven by lower contrast values for higher levels of ADHD severity (‘impulsivity’) in individuals with low levels of AUD (i.e. negative correlations). This has also been observed for the right middle temporal gyrus, pallidum, putamen and insula. In these regions, individuals with high levels of AUD also showed higher contrast values for higher levels of ADHD. See Table 3A and Figs. 2 to 5 for further details. Regarding the difference between low and high levels of ADHD severity (see Table 3B) interaction effects in the in the right middle and superior frontal gyrus, BA10, middle temporal gyrus, pallidum and thalamus, and bilateral lingual gyrus were driven by lower contrast values for higher levels of AUD severity in individuals with low levels of ADHD (i.e. negative correlations). This has also been observed for the right posterior cingulate gyrus, insula and inferior frontal gyrus. In these regions, individuals with high levels of ADHD additionally showed higher contrast values for higher levels of AUD severity. See Table 3B and Figs. 2 to 5 for more details. The exploratory group comparisons showed greater BOLD (blood oxygen level dependent) response in AUD + ADHD participants in this contrast (alcohol > neutral/scramble; incongruent > congruent) in the angular gyrus, insula, middle and posterior cingulate, middle, superior, and inferior frontal gyrus, precentral gyrus, supplementary motor area, and middle and superior temporal gyrus when compared to AUD participants (see supplementary Table 3 and supplementary Fig. 5) and in the insula, anterior, middle, and posterior cingulate, medial frontal gyrus and supplementary motor area when compared to ADHD participants (see supplementary Table 4 and supplementary Fig. 6).

**Table 3:** Pearson correlation analysis between aggregated brain activation (contrast “alcohol vs. neutral/scramble, incongruent vs. congruent”) and symptom load in ROIs with significant interaction effects between AUD and ADHD severity. A: Median split into low/high ADS; correlation with ADHD severity. B: Median split into low/high ADHD; correlation with AUD severity

**A**	**ROI**	**Low ADS (‘impulsivity’)** **corr. Coeff./ sign**	**High ADS (‘impulsivity’)** **corr. Coeff./ sign**	**Low ADS (‘overall’)** **corr. Coeff./ sign**	**High ADS (‘overall’)** **corr. Coeff./ sign**
	Right middle frontal gyrus	**−0.428/ .033**	0.359/ .085	−0.349/ .088	0.384/ .064
	Right thalamus	**−0.683/ .000 **	0.178/ .404	**−0.509/ .009**	0.261/ .217
	Right middle temporal gyrus	**−0.642/ .001**	0.364/ .080	**−0.604/ .001**	**0.406/ .049**
	Right BA10	**−0.458/ .021**	0.384/ .064	−0.341/ .095	0.382/ .066
	Right insula	**−0.486/ .014**	**0.429/ .037**	**−0.431/ .031**	**0.447/ .028**
	Left lingual gyrus	**−0.537/ .006**	0.252/ .235	−0.333/ .104	0.290/ .170
	Right lingual gyrus	**−0.566/ .003**	0.235/ .268	−0.354/ .083	0.275/ .193
	Right lingual gyrus	**−0.549/ .004**	0.335/ .110	**−0.510/ .009**	0.338/ .106
	Right superior frontal gyrus	**−0.462/ .020**	0.362/ .082	−0.380/ .061	0.380/ .067
	Right inferior frontal gyrus	**−0.575/ .003**	0.218/ .307	**−0.454/ .023**	0.321/ .126
	Right pallidum	**−0.586/ .002**	**0.418/ .042**	**−0.553/ .004**	**0.430/ .036**
	Right putamen	**−0.568/ .003**	**0.503/ .012**	**−0.481/ .015**	**0.525/ .008**
**B**	**ROI**	**Low ADHD (‘impulsivity’)** **corr. Coeff./ sign**	**High ADHD (‘impulsivity’)** **corr. Coeff./ sign**	**Low ADHD (‘overall’)** **corr. Coeff./ sign**	**High ADHD (‘overall’)** **corr. Coeff./ sign**
	Right middle frontal gyrus	**−0.690/ .000**	0.161/ .451	**−0.685/ .000**	0.274/ .196
	Right thalamus	**−0.515/ .008**	0.248/ .244	**−0.473/ .017**	0.231/ .278
	Right middle temporal gyrus	**−0.714/ .000**	0.279/ .187	**−0.680/ .000**	0.303/ .150
	Right BA10	**−0.670/ .000**	0.233/ .272	**−0.641/ .001**	0.301/ .153
	Right insula	−.0.386/ .057	**0.410/ .047**	−0.346/ .090	0.396/ .055
	Left lingual gyrus	**−0.526/ .007**	0.363/ .082	**−0.506/ .010**	0.395/ .056
	Right lingual gyrus	**−0.479/ .016**	0.304/ .149	**−0.462/ .020**	0.319/ .129
	Right lingual gyrus	**−0.511/ .009**	**0.493/ .014**	**−0.476/ .016**	**0.483/ .017**
	Right superior frontal gyrus	**−0.695/ .000**	0.184/ .388	**−0.687/ .000**	0.284/ .179
	Right inferior frontal gyrus	−0.351/ .085	0.391/ .059	−0.385/ .058	**0.442/ .031**
	Right pallidum	**−0.462/ .020**	0.312/ .138	**−0.515/ .008**	0.398/ .054
	Right putamen	−0.272/ .189	0.289/ .171	−0.316/ .124	0.360/ .084

**Fig. 6:**
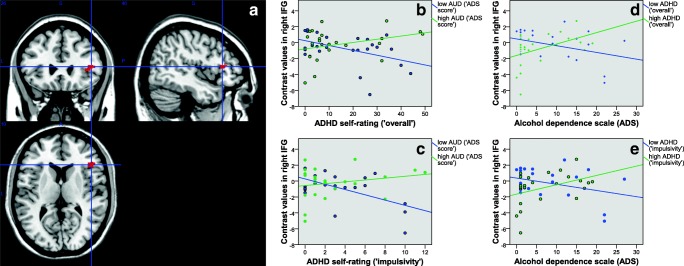
A: functional ROI mask of the right inferior frontal gyrus (IFG) for contrast value aggregation; B-E: association between AUD or ADHD severity and aggregated brain activation (contrast “alcohol vs. neutral/scramble, incongruent vs. congruent”; n = 49); B + C: median split of all participants into low and high AUD, association of brain activation with ADHD severity (B:‘overall’; C:‘impulsivity’); D + E: median split of all participants into low and high ADHD (D:‘overall’; E:‘impulsivity’), association of brain activation with AUD severity

## Discussion

ADHD prevalence rates are higher in the AUD population than in the general population (Daigre et al., 2015; Luderer et al., 2018; Reyes et al., 2016; Roncero et al., 2019). Both ADHD and AUD have been associated with deficits in inhibitory control (Barkley, 1997; Herman & Duka, 2019; Pedersen et al., 2016; Roberts et al., 2014; Smith et al., 2014; Wright et al., 2014). The aim of this study was to examine the effects of behavioral inhibition and cue-reactivity in individuals with either ADHD or AUD, healthy controls and in individuals with both ADHD and AUD and the corresponding interaction effects of task and disorder on neural activity. Our data showed a positive correlation between AUD severity and brain activity in reward-related regions, especially in individuals with high ADHD symptom load, which supports our first hypothesis. Our second hypothesis theorizing a negative association between prefrontal brain activity and ADHD severity in the presence of higher AUD symptom load could not be supported. This study is one of the first to be able to shed light on the combined effects of ADHD and AUD on inhibition and cue-reactivity. We used an experimental paradigm which mirrors a real-life situation more closely than most laboratory assessments: a hybrid task investigating the need for inhibition in an environment full of alcohol-associated stimuli (e.g. being asked at a party: ‘just have a beer already, will you?’). Regarding alcohol-associated cues, previous studies described increased cue related activity in the insula, striatum, the amygdala and frontal regions such as the ACC, medial PFC or OFC (Bach et al., 2020; Goldstein & Volkow, 2002; Heinz, Beck, Grusser, Grace, & Wrase, 2009; Vollstädt-Klein et al., 2012; Vollstädt-Klein et al., 2010). Our results suggest that salience attribution and cue-reactivity in response to alcohol cues is stronger in individuals with higher levels of ADHD severity. Further examination is needed to determine whether depleted cognitive resources might underlie this increased cue reactivity, as the current study did not reveal a significant negative correlation between prefrontal activity and ADHD severity. In such a model, individuals with higher levels of ADHD symptom load must divert cognitive effort away from cue-reactivity suppression in order to perform the parallel inhibition task.

Within the high AUD group, we observed increased activation within the right thalamus, pallidum, putamen (part of the DS), and insula as ADHD severity increased. These regions have all been previously implicated in mediating various aspects of the development and maintenance of addictive behaviors. The thalamus is, in general, seen as a hub serving multiple purposes. In addiction research, it plays a role in salience attribution and has been observed to show hyperactivation in the presence of drug cues but hypoactivation during response inhibition (Huang, Mitchell, Haber, Alia-Klein, & Goldstein, 2018). The pallidum has been observed to play a crucial role in motivation and therefore addictive behaviors, supported by various animal models (Farrell et al., 2019; Volkow & Morales, 2015). The DS, which also encompasses the putamen, is involved in habit formation, especially in the context of addiction (Volkow, Wang, Fowler, & Tomasi, 2012). It has also been shown that a shift from ventral to dorsal activation with respect to cue-reactivity takes place in heavy drinkers (Vollstädt-Klein et al., 2010). The right insula has been associated with the attention system. More precisely, it may mediate the coordination and evaluation of task performance during perceptional and response demands (Eckert et al., 2009). Additionally, the insula has been associated with craving (Naqvi, Gaznick, Tranel, & Bechara, 2014; Noël, Brevers, & Bechara, 2013).

In our study, the increased activity observed in these regions for high AUD/high ADHD individuals may be explained due to weaker inhibitory control over cue-reactivity: downregulation of this cue-reactivity seems to fail in the presence of alcohol-related stimuli – but only in the interaction condition (stimulus vs. congruency). An exhaustion of cognitive capacities through the interference effect of the task may have reduced the ability to inhibit alcohol-related neural reactivity. It has been previously shown that in adult ADHD, a decrease of activation occurs in inhibitory control regions during inhibition tasks (Hart et al., 2013). On the other hand, the higher activation in the insula in individuals with higher severity of AUD and ADHD may also represent a compensatory mechanism, since the insula is also responsible for self-awareness and interoception (Eckert et al., 2009). Striatal dopaminergic states (either hypo- or hyperdopaminergic) may also serve as a link between and common mechanistic explanation for AUD and ADHD as well as impulsivity and cue reactivity (Hansson et al., 2019; Hirth et al., 2016; Volkow et al., 2009; Wiers et al., 2018), but further studies investigation this potential relation are needed.

In individuals with low AUD symptom load, higher levels of ADHD symptom load lead to a decrease in activity in subcortical, reward-related regions (thalamus, pallidum, putamen, insula). This is in line with previous findings on ADHD and the reward system. Stark et al. (2011) observed a negative correlation between the neural response in the reward system and ADHD severity. This has been explained within the framework of a deficit in the reward system (Stark et al., 2011). Regarding prefrontal control regions in the low AUD group, higher ADHD levels lead to lower activity (inferior, middle, superior frontal gyrus, middle temporal gyrus, lingual gyri) since alcohol stimuli do not play a relevant role as a distractor in these individuals. Lower activity in inhibitory-related regions has been consistently observed in ADHD patients and has been interpreted as a low inhibitory control in ADHD (Cortese et al., 2012; Hart et al., 2013).

In individuals with high ADHD symptom load (‘impulsivity’), a positive correlation between AUD severity and brain activity in reward-related regions has also been observed (insula). Here, the same explanation may be applied: The cognitive capacity of these individuals has already been used up for the demanding interaction task. Therefore, weaker cognitive control remains for suppressing cue-reactivity in reward-related regions, which leads to hyperactivation, representing a potentiation of impaired inhibitory control in individuals suffering from both AUD and ADHD. However, this mechanistic explanation needs further examination, since the current study does not provide conclusive evidence for this claim. In fact, we found a positive correlation for high ADHD individuals between brain activity in the right thalamus, right inferior frontal gyrus, and right lingual gyrus and AUD symptom load. This does not necessarily contradict our suggestion that the previously identified increased cue reactivity for high AUD/high ADHD individuals might be a result of depleted cognitive resources. Instead, hyperactivation of these inhibitory and attention-related regions in high ADHD/high AUD individuals could reflect precisely the ‘taxing’ cognitive effect exerted on high ADHD individuals by a comorbid AUD diagnosis. The lingual gyrus is part of the primary visual area and is related to perception and recognition of familiar scenes or encoding of complex pictures (Machielsen, Rombouts, Barkhof, Scheltens, & Witter, 2000). Benedek et al. (2016) have found that internally directed attention was associated with an increased activation of the bilateral lingual gyrus, which they interpreted to reflect increased visual imagery during internal attention conditions. Another study has found increased activation of the lingual gyrus specifically in an overlapping dual-task set-up, where two different stimuli were presented in rapid succession and required the participant to respond to each separately (Schubert & Szameitat, 2003). This suggests a role for the lingual gyrus in mediating between competing demands on our attention. With respect to our results, the increased activation of the lingual gyrus might reflect the heightened distractor potential of alcohol cues for high ADHD/high AUD individuals while performing the hybrid task. The inferior frontal gyrus has also been implicated in attentional control, as well as inhibition, in general (Hampshire, Chamberlain, Monti, Duncan, & Owen, 2010). Furthermore, our observations are in line with previous findings that showed hypoactivation in individuals with ADHD during cognitively demanding tasks, but hyperactivation to drug-related stimuli (Brewer, Garrison, & Whitfield-Gabrieli, 2013; Brody et al., 2007; Leech & Sharp, 2014).

In individuals with low ADHD symptom load, a negative correlation between AUD severity and brain activation in reward-related regions was observed (pallidum). Prefrontal cognitive control may be exercised in these individuals in order to succeed in our combined task. The interference effect can be ‘handled’ by individuals only suffering from AUD, but not those with a concurrent ADHD diagnosis. In reward-related regions, a weaker reactivity to alcoholic stimuli can be explained as a result of higher cognitive control over this neural response. Further, previous studies observed a decrease of neural reactivity to substance-related cues in subcortical reward regions that was, amongst others, associated with SUD severity (Jasinska, Stein, Kaiser, Naumer, & Yalachkov, 2014; Vollstädt-Klein et al., 2011; Vollstädt-Klein et al., 2010).

On a behavioral level, no significant differences were found between groups. Also, there were no significant results regarding the relationship between RT and interference effects and AUD and/ or ADHD severity. This has been observed previously in other studies using different paradigms in individuals with either ADHD or AUD (Braet et al., 2011; Stark et al., 2011; Zilverstand et al., 2018). Results in our study may also stand for successful compensation in the group with high symptom load in both AUD and ADHD. Highly affected individuals may still be able to obtain results that are similar to those of the other groups within this short laboratory experiment. The descriptive findings of increased activation in the observed control areas in comorbid individuals might represent a compensation of inhibitory deficits. Assuming rather subtle differences between groups on the behavioral level, it might be legitimate to extrapolate population effects from small fMRI sample sizes based on the fact that effects on the neural level are stronger, as suggested by Berns and Moore (2012), amongst others.

Taken together, comorbidity of AUD with ADHD in comparison to AUD only seems to additionally decrease the ability to ignore alcohol cues, therefore affecting interference inhibition. The observed higher ADHD symptom severity in comorbid individuals in this sample, especially impulsivity, might also resemble a predisposing factor for developing a comorbidity of AUD in ADHD. As proposed in other studies, ADHD individuals are more susceptible to developing AUD (Charach et al., 2011; Estévez-Lamorte et al., 2019; Estévez et al., 2015; Lee et al., 2011; Wilens & Morrison, 2011). Increased impulsivity (Pedersen et al., 2016; Roberts et al., 2014) and decreased inhibitory control (Smith et al., 2014) have been associated with the development of AUD. Especially impulsivity has been demonstrated to mediate the relationship of adult alcohol problems after childhood ADHD (Pedersen et al., 2016). Our study supports the notion that higher levels of ADHD symptoms predispose for higher relapse rates and possibly the development and maintenance of AUD. Our results further stress the importance of reducing the attentional bias and cue-provoked craving in AUD individuals with or without comorbidity. The conclusive treatment model based on increased alcohol cue-reactivity in AUD is cue-exposure therapy (CET) and has been demonstrated to reduce cue-provoked craving (Unrod et al., 2013). A pharmacological approach to reduce cue-reactivity in AUD is the opioid antagonist naltrexone: Mann et al. (2014) demonstrated that AUD patients with high cue-reactivity to alcohol images profited more from the treatment with naltrexone than patients with lower cue-reactivity. The effectiveness of these treatments should be assessed in a sample of comorbid AUD and ADHD patients to determine whether they are equally effective in this subgroup as in AUD patients. Comorbid individuals tend to show an earlier onset of abuse, greater SUD comorbidity, as well as higher rates of substance abuse, suicide attempts, hospitalization, and depression (Arias et al., 2008; Huntley et al., 2012; Luderer et al., 2018). As stated by Huntley et al. (2012), the comorbidity of SUD and ADHD is not only “a source of additional impairment to patients” but also represents “an increased burden on clinical services”. The results in this study further support the idea that AUD and ADHD work synergistically, i.e. high symptom load in both domains results in more significant impairments, not just reflected in clinical observations but on a fundamental neural mechanistic level. It is therefore crucial that ADHD is identified as soon as possible during SUD treatment. As recommended by an international consensus group, an instrument to identify ADHD should be incorporated into the standard assessment protocol at treatment onset for SUD (Crunelle et al., 2018).

## Limitations

During recruitment of the participants and study execution, several complications were observed, some originating from the nature of the patient’s specific disorders. Even though all participants were carefully selected according to the inclusion criteria, some reported medication intake retrospectively in the questionnaire, resulting in noncompliance with inclusion criteria and subsequent exclusion from data analysis. Furthermore, the motivation to participate was observed to vary. Some participants did not return the questionnaire at all. In addition, some patients were not able to complete the fMRI experiment or showed heavy movement in the scanner. Taken together, this explains the high number of drop-outs. Furthermore, all patients were male, potentially limiting the generalizability of our results. Lastly, the four groups were not evenly numbered. The AUD + ADHD group, in particular, resulted in only six analyzable participants. Therefore, group comparisons are only reported in the supplementary material, and represent a more explorative level (using a liberal threshold). The primary analysis was conducted using a dimensional analysis approach. To further explore the interaction effects of the multilayered data (stimuli vs. congruency and AUD vs. ADHD severity) we used median-splits for AUD and ADHD severity, respectively.

## Conclusion

Functional alterations in reward and cognitive control related regions were observed to correlate with AUD symptom severity and were also affected by the interaction of AUD and ADHD severity - despite the absence of differences on the behavioral level of inhibition. This discrepancy may indicate a compensation of inhibitory deficits by increased neural activation, but could also be explained by increased sensitivity to detection of neural alterations versus behavioral responses. Increased neural activation in the combined task was observed in reward related, subcortical regions with increased symptom severity of AUD and ADHD in high ADHD and high AUD individuals, respectively. This suggests that AUD + ADHD participants are more severely affected than participants with only one of the disorders. The strongest interference effect for alcohol vs. neutral cues in this group might be a result of deficits in ignoring alcohol distractors due to reduced inhibition ability, which suggests that treatment of ADHD might be highly relevant in these patients. Furthermore, this patient group might be a candidate for interventions that aim to reduce cue-reactivity. Taken together, the results of our study suggest that AUD patients should be screened for ADHD, as identification of ADHD is highly relevant for individually adapted treatment and may result in improved relapse prevention.

## Electronic supplementary material

ESM 1 (DOCX 1378 kb)
