# Supplementary material for: Interaction between behavioral inhibition and neural alcohol cue-reactivity in ADHD and alcohol use disorder
Source: Psychopharmacology (Berl). 2020 Apr 13;237(6):1691–707. doi: 10.1007/s00213-020-05492-1 (PMC7239811; doi:10.1007/s00213-020-05492-1)
Supplement: Supplementary file 1 — (DOCX 1378 kb) [file 213_2020_5492_MOESM1_ESM.docx]

**Interaction between behavioral inhibition and neural alcohol cue-reactivity in ADHD and alcohol use disorder**

Supplementary Material

**SUPPLEMENTARY Figures**

**Supplementary Figure 1**

**
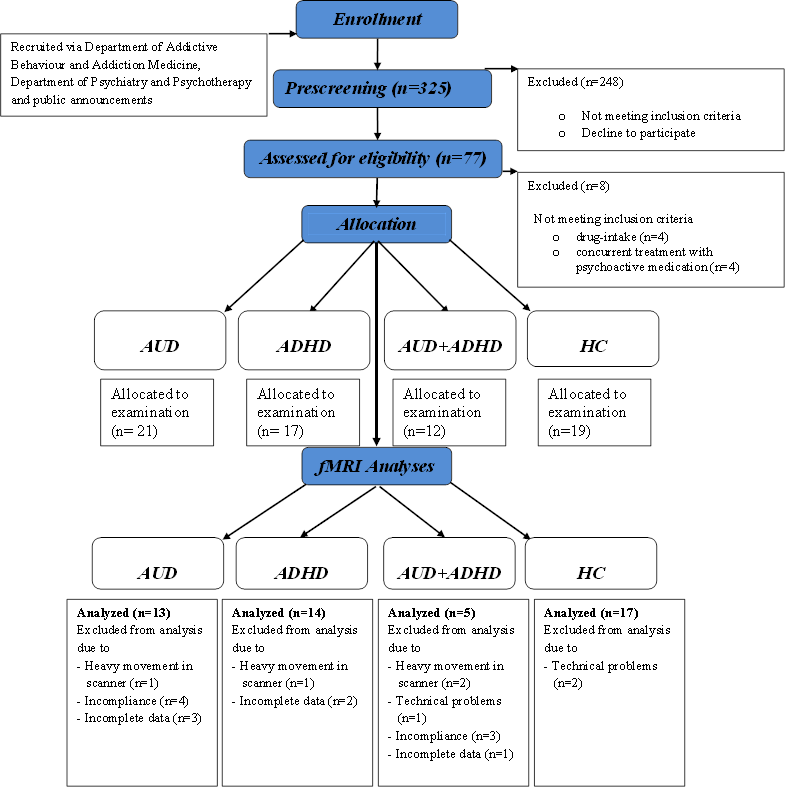
**

**Supplementary Figure 2**


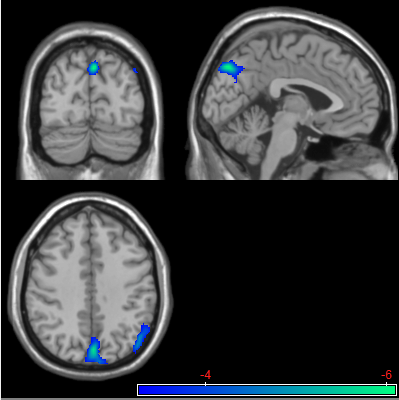


**Supplementary Figure 3**


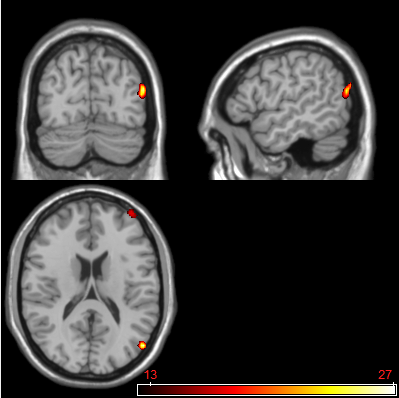


**Supplementary Figure 4**


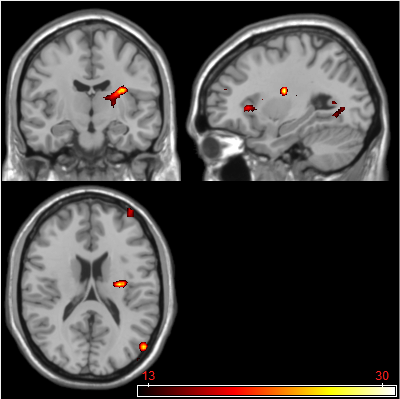

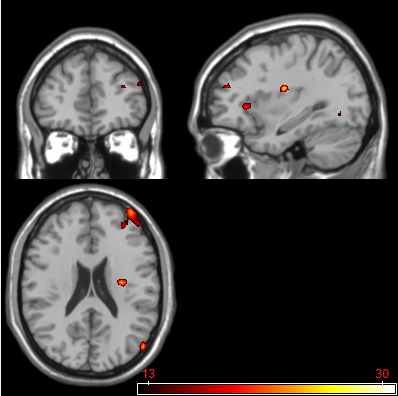


**Supplementary Figure 5**

**
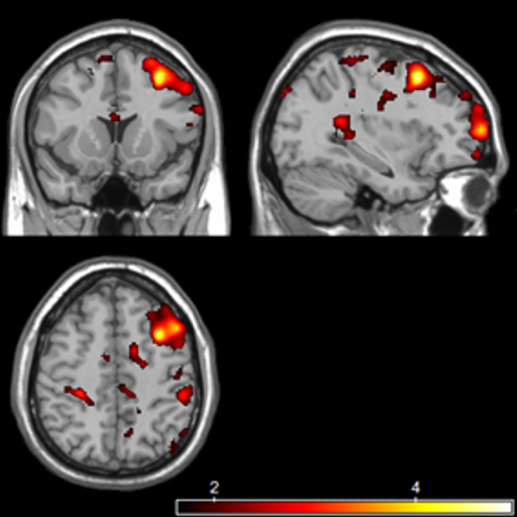
**

**Supplementary Figure 6**

**
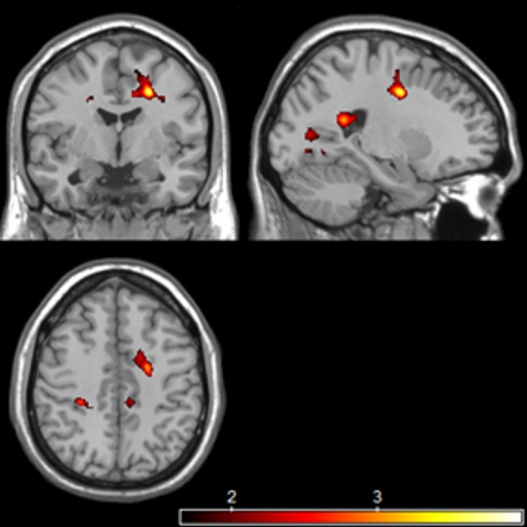
**

**SUPPLEMENTARY TABLES**

**Supplementary Table 1
Inclusion and exclusion criteria**

| Inclusion criteria | Exclusion criteria |
| --- | --- |
| Man or woman | Other Axis I psychiatric diagnoses |
| Age between 18 and 66 years | Psychotropic medication within the last 3 days |
| Alcohol use disorder according to DSM-5 | Intake of drugs (other than alcohol or tobacco) within the last 3 weeks or cocaine, amphetamine, opioid dependence lifetime |
| ADHD according to DSM-5 | Severe physical illness |
| Abstinence from alcohol (5 days till 6 weeks prior to study inclusion)  Normal or corrected to normal vision | Severe withdrawal symptoms (CIWA-Ar > 4; Sullivan et al. 1989) at the time of MRI investigation  Pregnancy |
| Signed written informed consent | Therapy with methylphenidate within the last 8 weeks |
|  | Common exclusion criteria for MRI (e.g. metal, claustrophobia) |
|  | Neurological disorders, history of brain injury |

| **Supplementary Table 2**  **A:** Mean (SD) of behavioral data of all participants (n=49), segregated by diagnosis. No significant group differences were observed. **B+C**: Pearson correlation analysis between behavioral measures (reaction times and interference effects) and symptom load. **B**: Mediansplit of all individuals into low/high ADS; correlation with ADHD severity. **C**: Mediansplit of all individuals into low/high ADHD; correlation with AUD severity. | | | | | | | | |
| --- | --- | --- | --- | --- | --- | --- | --- | --- |
| **A** | **AUD**  **Mean(SD)** | | **ADHD**  **Mean(SD)** | | **AUD+ADHD**  **Mean(SD)** | | **Healthy controls**  **Mean(SD)** | |
| **Reaction time [ms]**  overall | 669 (75) | | 620 (105) | | 638 (90) | | 637 (128) | |
| **Reaction time [ms]**  alcohol - neutral | -5 (25) | | -14 (24) | | -17 (12) | | -20 (16) | |
| **Interference effect [ms]** | 48 (28) | | 37 (24) | | 47 (32) | | 46 (26) | |
| **Interference effect [ms]**  alcohol - neutral | 10 (34) | | -9 (37) | | 23 (32) | | 4 (59) | |
| **B** | **Low ADS (‘impulsivity’)**  **corr. coeff./ sign** | **High ADS (‘impulsivity’)**  **corr. coeff./ sign** | | **Low ADS (‘overall’)**  **corr. coeff./ sign** | | **High ADS (‘overall’)**  **corr. coeff./ sign** | |  |
| **Reaction time [ms]**  overall | -0.065/ .759 | -0.073/ .736 | | -0.241/ .246 | | -0.087/ .687 | |  |
| **Reaction time [ms]**  alcohol - neutral | 0.116/ .582 | -0.189/ .377 | | 0.117/ .578 | | -.238/ .262 | |  |
| **Interference effect [ms]** | **-0.430/ .032** | -.072/ .740 | | -0.238/ .251 | | -0.138/ .521 | |  |
| **Interference effect [ms]**  alcohol - neutral | 0.115/ .584 | -0.024/ .912 | | -0.040/ .849 | | 0.013/ .954 | |  |
| **C** | **Low ADHD (‘impulsivity’)**  **corr. coeff./ sign** | **High ADHD (‘impulsivity’)**  **corr. coeff./ sign** | | **Low ADHD (‘overall’)**  **corr. coeff./ sign** | | **High ADHD (‘overall’)**  **corr. coeff./ sign** | |  |
| **Reaction time [ms]**  overall | 0.126/ .548 | 0.016/ .940 | | 0.148/ .481 | | -0.029/ .892 | |  |
| **Reaction time [ms]**  alcohol - neutral | 0.322/ .105 | 0.132/ .539 | | **0.429/ .032** | | -0.055/ .798 | |  |
| **Interference effect [ms]** | -0.225/ .280 | 0.098/ .648 | | -0.152/ .467 | | -0.045/ .834 | |  |
| **Interference effect [ms]**  alcohol - neutral | -0.004/ .985 | 0.305/ .147 | | 0.029/ .891 | | 0.218/ .306 | |  |

**Supplementary Table 3**

Brain areas in which BOLD response for the contrast “alcohol vs. neutral/scramble, incongruent vs. congruent” was significantly larger in AUD+ADHD compared to AUD

| Side | Lobe | Brain Areas | Brodmann Area | Cluster Size | MNI Coordinates | | | t_maximum_ | |  |
| --- | --- | --- | --- | --- | --- | --- | --- | --- | --- | --- |
| Right | Frontal | Middle frontal gyrus, superior frontal gyrus, precentral gyrus, supplementary motor area, inferior frontal gyrus | 9, 10, 46, 6, 4, 8 | 16288 | 32 | 14 | 48 | | 4.92 | |
| Left | Frontal | Supplementary motor area, superior frontal gyrus, middle frontal gyrus, precentral gyrus | 6, 4, 8, 9 |  |  |  |  | |  | |
| Right | Parietal | Supramarginal gyrus, postcentral gyrus, angular gyrus, precuneus, superior parietal lobule, inferior parietal lobule | 40, 3/2, 39, 7 |  |  |  |  | |  | |
| Left | Parietal | Postcentral gyrus, paracentral lobule, precuneus, superior parietal lobule, inferior parietal lobule | 3/2, 5, 7 |  |  |  |  | |  | |
| Right | Limbic | Middle cingulate, posterior cingulate | 23, 31, 24 |  |  |  |  | |  | |
| Left | Limbic | Middle cingulate, posterior cingulate | 23, 31, 24 |  |  |  |  | |  | |
| Right | Temporal | Middle temporal gyrus | 21 |  |  |  |  | |  | |
| Left | Temporal | Superior temporal gyrus | 38 |  |  |  |  | |  | |
| Right | Occipital | Middle occipital gyrus, superior occipital gyrus, calcarine sulcus | 19, 18 |  |  |  |  | |  | |
| Left | Occipital | Calcarine sulcus | 18 |  |  |  |  | |  | |
| L/R |  | Insula | 13 |  |  |  |  | |  | |

*Note.* MNI = Montreal Neurological Institute. Combined voxel-wise- [*P* < 0.05] and cluster-extent threshold [*k* >= 1508 voxel], corresponding to *p*FWE < 0.05

| **Supplementary Table 4**  Brain areas in which BOLD response for the contrast ‘alcohol > neutral/scramble, incongruent - congruent’ was significantly larger in AUD+ADHD compared to ADHD | | | | | | | |
| --- | --- | --- | --- | --- | --- | --- | --- |
| Side | Lobe | Brain Areas | Brodmann Area | Cluster Size | MNI Coordinates | t_maximum_ | |
| Right  Left | Frontal  Frontal | Supplementary motor cortex, medial frontal gyrus  Medial frontal gyrus | 6, 8  8 | 2576 | 22 -4 40 | 3.97 |  |
| Right | Occipital | Calcarine sulcus, middle occipital gyrus, fusiform gyrus, lingual gyrus, cuneus | 18, 19 |  |  |  |  |
| Right | Limbic | Anterior cingulate, middle cingulate, posterior cingulate | 24, 23 |  |  |  |  |
| Left | Limbic | Anterior cingulate, posterior cingulate | 24, 23 |  |  |  |  |
| R/L |  | Insula |  |  |  |  |  |

*Note.* MNI = Montreal Neurological Institute. Combined voxel-wise- [*P* < 0.05] and cluster-extent threshold [*k* >= 1508 voxel], corresponding to *p*FWE < 0.05.

**SUPPLEMENTARY FIGURE LEGENDS**

**Supplementary Figure 1** Consort flow chart displaying inclusion and allocation processes. N=49 individuals were included in the final fMRI analyses.

**Supplementary Figure 2** Negative correlation of brain activation with AUD severity (assessed via alcohol dependency scale) during incongruent trials compared to congruent trials in which alcohol stimuli were presented (functional overlay at: 2/-78/40; n=49; combined voxel-wise [P < 0.001] and cluster-extent threshold [k >= 151 voxel], corresponding to pFWE < 0.05).

**Supplementary Figure 3** Brain activation for the interaction of AUD and ADHD severity (assessed via alcohol dependency scale and ‘overall’ factor of ADHD self-report scale) during incongruent trials compared to congruent trials in which alcohol stimuli were presented (functional overlay at: 52/-74/18; n=49; combined voxel-wise [P < 0.001] and cluster-extent threshold [k >= 151 voxel], corresponding to pFWE < 0.05).

**Supplementary Figure 4** Brain activation for the interaction of AUD and ADHD severity (assessed via alcohol dependency scale and ‘impulsivity’ factor of ADHD self-report scale) during incongruent trials compared to congruent trials in which alcohol stimuli were presented (left: functional overlay at 32/44/2; right: functional overlay at 28/-12/18; n=49; combined voxel-wise [P < 0.001] and cluster-extent threshold [k >= 151 voxel], corresponding to pFWE < 0.05).

**Supplementary Figure 5** Increased brain activation in AUD+ADHD (n = 6) compared to AUD (n = 16) during incongruent trials compared to congruent trials in which alcohol stimuli were presented (combined voxel-wise [P < 0.05] and cluster-extent threshold [k >= 1508 voxel], corresponding to pFWE < 0.05).

**Supplementary Figure 6** Increased brain activation in AUD+ADHD (n = 6) compared to ADHD (n = 16) during incongruent trials compared to congruent trials in which alcohol stimuli were presented (combined voxel-wise [P < 0.05] and cluster-extent threshold [k >= 1508 voxel], corresponding to pFWE < 0.05).
